# Metachrony drives effective mucociliary transport via a calcium-dependent mechanism

**DOI:** 10.1152/ajplung.00392.2023

**Published:** 2024-06-11

**Authors:** Jacelyn E. Peabody Lever, K. Brett Turner, Courtney M. Fernandez, Hui Min Leung, Shah Saddad Hussain, Ren-Jay Shei, Vivian Y. Lin, Susan E. Birket, Kengyeh K. Chu, Guillermo J. Tearney, Steven M. Rowe, George M. Solomon

**Affiliations:** ^1^Division of Pulmonary and Critical Care Medicine, Department of Medicine, University of Alabama at Birmingham, Birmingham, Alabama, United States; ^2^Medical Scientist Training Program, University of Alabama at Birmingham, Birmingham, Alabama, United States; ^3^Division of Pulmonary Medicine, Department of Pediatrics, University of Alabama at Birmingham, Birmingham, Alabama, United States; ^4^Department of Anesthesiology and Perioperative Medicine, University of Alabama at Birmingham, Birmingham, Alabama, United States; ^5^Gregory Fleming James Cystic Fibrosis Research Center, University of Alabama at Birmingham, Birmingham, Alabama, United States; ^6^Wellman Center for Photomedicine, Massachusetts General Hospital, Boston, Massachusetts, United States; ^7^Harvard Medical School, Boston, Massachusetts, United States; ^8^Harvard-MIT Division of Health Sciences and Technology, Cambridge, Massachusetts, United States; ^9^Department of Pathology, Harvard Medical School and Massachusetts General Hospital, Boston, Massachusetts, United States

**Keywords:** airway physiology, calcium signaling, cilia, metachrony, mucociliary transport

## Abstract

The mucociliary transport apparatus is critical for maintaining lung health via the coordinated movement of cilia to clear mucus and particulates. A metachronal wave propagates across the epithelium when cilia on adjacent multiciliated cells beat slightly out of phase along the proximal-distal axis of the airways in alignment with anatomically directed mucociliary clearance. We hypothesized that metachrony optimizes mucociliary transport (MCT) and that disruptions of calcium signaling would abolish metachrony and decrease MCT. We imaged bronchi from human explants and ferret tracheae using micro-optical coherence tomography (µOCT) to evaluate airway surface liquid depth (ASL), periciliary liquid depth (PCL), cilia beat frequency (CBF), MCT, and metachrony in situ. We developed statistical models that included covariates of MCT. Ferret tracheae were treated with BAPTA-AM (chelator of intracellular Ca^2+^), lanthanum chloride (nonpermeable Ca^2+ ^channel competitive antagonist), and repaglinide (inhibitor of calaxin) to test calcium dependence of metachrony. We demonstrated that metachrony contributes to mucociliary transport of human and ferret airways. MCT was augmented in regions of metachrony compared with nonmetachronous regions by 48.1%, *P* = 0.0009 or 47.5%, *P* < 0.0020 in humans and ferrets, respectively. PCL and metachrony were independent contributors to MCT rate in humans; ASL, CBF, and metachrony contribute to ferret MCT rates. Metachrony can be disrupted by interference with calcium signaling including intracellular, mechanosensitive channels, and calaxin. Our results support that the presence of metachrony augments MCT in a calcium-dependent mechanism.

**NEW & NOTEWORTHY** We developed a novel imaging-based analysis to detect coordination of ciliary motion and optimal coordination, a process called metachrony. We found that metachrony is key to the optimization of ciliary-mediated mucus transport in both ferret and human tracheal tissue. This process appears to be regulated through calcium-dependent mechanisms. This study demonstrates the capacity to measure a key feature of ciliary coordination that may be important in genetic and acquired disorders of ciliary function.

## Graphical abstract

**Figure:**
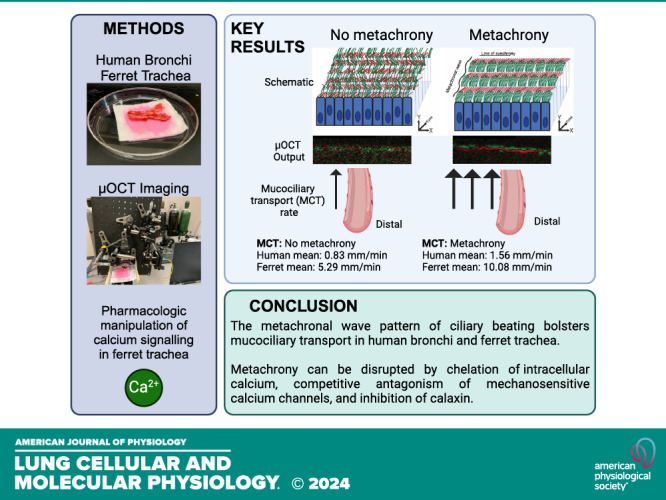

## INTRODUCTION

The respiratory epithelium plays a significant role in defense against inhaled particulates and pathogens to maintain lung health via mucociliary clearance (1). Secretory cells release compounds crucial for airway defense, including mucins and liquid, forming the airway surface liquid (ASL) or mucus gel layer. This layer, along with the periciliary liquid (PCL), influences mucociliary transport (MCT). The components of the airway epithelium maintain hydration and preserve proper mucus physiochemistry to propel foreign material out of the airways (2). Derangement of this innate defense disrupts clearance from the airways and precipitates severe lung disease (3–5).

Motile cilia beat in a pattern comprising a highly coordinated effective and recovery stroke (6). Defective ciliary motion or cilia beat frequency (CBF) reduces mucociliary transport (MCT). However, the role of intercoordination of cilia in generating and maintaining MCT is poorly understood. Disordered mucociliary clearance attributed to acquired or genetic abnormalities of ciliary structure and motion in pathological mucus contributes to cystic fibrosis (CF), primary ciliary dyskinesia (PCD), chronic obstructive lung disease (COPD), non-CF bronchiectasis, and asthma (3, 7).

Metachrony is a complex mechanism that is characterized by coordinated motion among adjacent cilia on multiciliated cells (8, 9). Metachronal beating appears as a propagating wave of successive and consistent asynchronous ciliary movement, allowing successive cilia to propel mucus along conducting airways in the direction of mucociliary transport. Metachrony has been hypothesized to help overcome the viscoelastic forces required for mucus propulsion (10–12), such that there is more particle displacement with the same ciliary force due to the power of a metachronal wave. Phase shifts between cilium and metachronal waves have been observed in different mucociliary culture preparations (13–17) as well as intact sheep and dog tracheae (18). Mathematical models have demonstrated the dependence of metachronal wave properties on cilia characteristics (19–21). However, mechanistic studies of metachrony have been limited to invertebrate model systems or artificial magnetic cilia (2, 8, 22), thus its importance in human mucociliary clearance in which cilia interact in a coordinated fashion presents a knowledge gap.

It has been shown that local calcium signaling and hydrodynamic forces physically transmit and contribute to metachronal waves in motile cilia. One possibility is that metachrony results in hydrodynamic coupling between cilia and that this coordinated ciliary motion is regulated by intra- and extracellular calcium concentrations (10, 23–26).

We investigated MCT rates in ciliated epithelium with and without metachronal beating using micro-optical coherence tomography (µOCT). μOCT is an imaging modality that we developed to conduct real-time, noninvasive imaging of the epithelial surface of living airways at subcellular resolution (27). The technique allows for simultaneous in situ evaluation of multiple parameters of the functional airway microanatomy, including measures of airway hydration (ASL, PCL depths), ciliary function (CBF), and MCT (1, 6, 22, 27–29). The biological significance of ASL, PCL, CBF, and MCT and their visualization has been reviewed elsewhere (6, 29).

Here, we developed a method to detect and measure metachrony to evaluate its impact on MCT in bronchi from human explants and excised ferret trachea. We hypothesized that in intact tissue capable of being measured with µOCT, metachrony would enhance MCT in a calcium-dependent fashion. Furthermore, disruptions of calcium signaling would abolish metachrony and lead to impaired MCT, disproportionate to effects on ciliary beating or other covariates of MCT.

## METHODS

### Human Bronchial Tissue Procurement

Bronchial tissue samples were obtained from lung explants from failed donors without a history of respiratory disease. Subjects provided written informed consent before the collection of human tissues, and University of Alabama at Birmingham Institutional Review Board approved all tissues. Human bronchial tissue samples were placed in DMEM with 100 mg/mL ceftazidime, 80 mg/mL tobramycin, and 1.25 mg/mL amphotericin B. Bronchial samples were cut longitudinally along the posterior trachealis muscle to expose the luminal airway epithelial surface, then cleared of excessive endogenous mucus or sputum. Tissues were incubated at physiological conditions (37°C, 5% CO_2_, 100% humidity) for ∼30 min before µOCT imaging.

### Ferret Models

Wild-type adult ferrets aged 22–26 wk were obtained from Marshall Biosciences. University of Alabama at Birmingham Institutional Animal Care and Use Committee approved all animal studies. Animals were anesthetized with a formulation containing dexmedetomidine (0.08–0.2 mg/kg, im) and ketamine (2.5–5 mg/kg, im) and then euthanized by exsanguination. Tracheae were excised for µOCT. Tracheas were dissected into 1.5–2 cm segments and placed onto gauze saturated with DMEM/F12 media (LifeTechnologies). Segments were then dissected longitudinally along the trachealis muscle on the posterior side to expose the luminal epithelial surface and incubated for ∼30 min under physiological conditions (37°C, 5% CO_2_, 100% humidity) in an environmental chamber (TempModule S1, PeCon).

### Pharmacological Modulation

After images were obtained in the baseline condition, ferret tissue segments were treated basolaterally (Fig. 4) with agents to modulate calcium concentration, mechanosensitive signaling, and calcium-dependent signaling (30–32). μOCT imaging was then conducted 30 min after incubation with the respective compound. To depress intracellular calcium, BAPTA-AM (100 μM, Sigma Aldrich) was applied. BAPTA-AM is a cell-permeant chelator that is highly selective for calcium and less sensitive to pH than other chelators. Mechanosensitive calcium channels were inhibited with lanthanum (III) chloride heptahydrate (1 mM, Sigma Aldrich) to explore the potential for mechanosensitive propagation of metachrony. Repaglinide (150 mM, Sigma Aldrich) was applied to inhibit calaxin and alter dynein motor protein function, which we hypothesized could affect calcium-dependent propagation of ciliary motion. Tracheae were maintained in physiological conditions (37°C, 5% CO_2_, 100% humidity) in a tissue culture incubator.

### µOCT Imaging and Quantification of ASL, PCL, CBF, MCT, and Metachrony

Functional assessments of the tracheal tissue explants were performed to assess airway surface liquid (ASL), periciliary liquid (PCL) depth, cilia beat frequency (CBF), and mucociliary transport rate (MCT) using µOCT as previously described (22, 27, 28, 33) in an environmental chamber with a temperature regulatory device (Incubator PM 2000 RBT with TempModule S, Carl Zeiss, Thornwood, NY). Briefly, µOCT is an interferometric imaging modality that generates a cross-sectional image with a subcellular resolution of ∼1–3 µm. Specifically, acquisition speed was set at 20,480 Hz A-line rates to yield 100 frames per second, with 240 A-lines per frame in the linear scan; playback speed of 40 fps. Images were recorded at 5–10 ROI per piece of tissue (each ROI is 240 μm wide) per condition on the anterior surface of the mid-trachea where there are adequate and consistent numbers of ciliated cells. Images were recorded systematically in a proximal to distal fashion along the luminal surface of the anterior mucosal surface of the mid-trachea with the optical beam scanned along the longitudinal direction as previously described (22, 27, 28, 33). All tissues were imaged with the beam directly perpendicular to the long axis of the trachea with tracheal tissue measured in the caudal to cranial direction so that the active ciliary plane was coincident with the µOCT beam; the beam was oriented perpendicular to the long axis of the trachea with MCT in the caudal to cranial direction. All image analysis was performed using ImageJ and MATLAB R2018. Our analysis of ciliary movement and metachrony using µOCT relies on the frequency distribution of light waves to detect the relative state of ciliary activity.

Airway surface liquid (ASL) and periciliary layer (PCL) depths were measured directly as previously described (22, 27, 28, 33). MCT rate was calculated based on the average slope of the distance per time of particulates in the mucus over several frames in the ASL region as previously described (22, 27, 28, 33). CBF was determined by temporally high-pass filtering the images using 0.6 Hz as the reference to remove low-frequency vibrations, computing the Fourier power spectrum for all 3×3 pixel subregions in the image, selecting the subregions ranking highest in peak sharpness, defined as the peak power density divided by the total power density (22, 27, 28, 33). Measurements from up to 10 subregions were aggregated for each ROI automatically, with values corresponding to spurious peaks manually rejected in cases of abnormal power spectral density appearance, such as multiple peak frequencies in one subregion.

**Figure 1.**
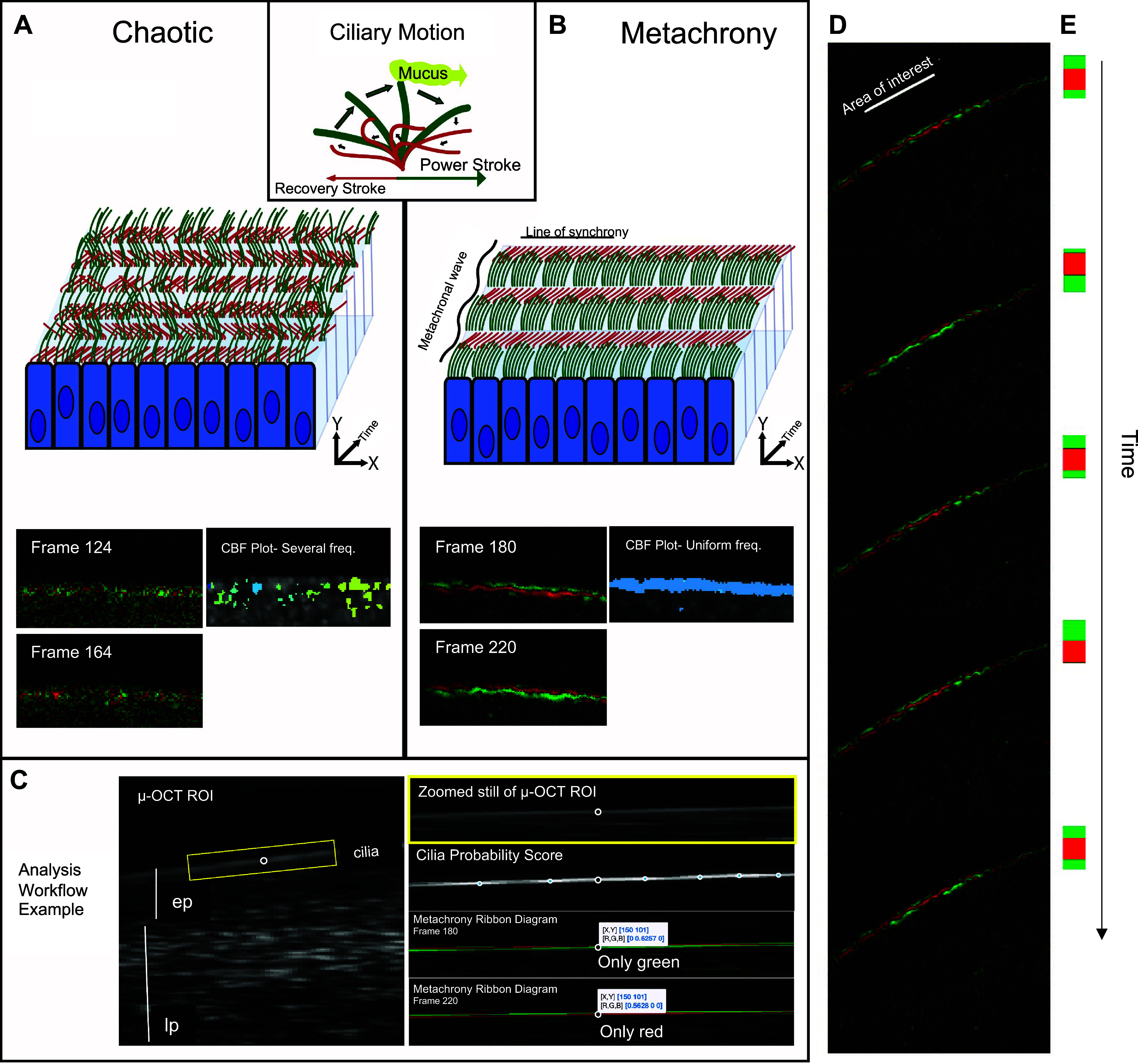
Ciliary motion in chaos and metachrony. Cilia beating is characterized by power and recover strokes (*inset*); green and red, respectively. Metachrony was defined when two factors were present in the ROI: *1*) at least 25% of the epithelial surface demonstrated a line of synchronous beating as evidenced by linear monochromatic ribbons, and *2*) these linear ribbons alternated between phases when analyzed over time. In contrast, areas without synchrony (i.e. red and green speckling) or coordinated phase shifts over time were non-metachronous. *A*: schematic of cilia beating chaotically, without coordination with neighboring ciliated cells. Representative Matlab ribbon diagram outputs from µOCT in human bronchi demonstrating the same area of the epithelium, 40 frames apart. Note random speckling of green and red and several frequencies present in the CBF plot. *B*: schematic of cilia beating in metachrony, defined in two dimensions by a line of synchronization and a perpendicular line of metachrony over the ciliary plane. Note the line of synchrony sliding from red to green in the ribbon diagram and the uniform CBF frequency. *C*: example of metachrony analysis workflow. First, user defines region of ciliation (yellow box) in the cross-sectional µOCT image; the distinct refractive properties of the epithelium (ep) and lamina propria (lp) are useful anatomic landmarks. Regions exhibiting oscillatory behavior receive a cilia probability score using the Matlab script and are denoted by a circle in the Cilia Probability Score output. The frequency of the peak amplitude in the temporal Fourier transform of the probable cilium is used to determine cilia beat frequency (CBF Plot shown in *A* and *B*). Ribbon diagrams are generated subsequently, which are made by binning distinct oscillatory regions into red or green based on the relative position of cilia. Note in frame 180, at [*X*,*Y*] coordinates [150, 101], the [red, green, blue] values are [0, 0.6257, 0], indicating the cilia are only green (power stroke). In the following panel, frame 220 is shown at the same location [150, 101], but now the [red, green, blue] values are [0.5628, 0, 0], indicating the cilia are only red (recovery stroke) 40 frames later. *D*: the ribbon diagram demonstrates how a metachronal wave is propagated across the epithelium when cilia on adjacent multiciliate cells beat in this coordinated phase shift in space and time (*E*).

Metachrony was measured by phase comparison across the epithelium, including the presence and proportion of metachrony (metachrony length/epithelial surface length, measured in ImageJ) per region of interest (see Fig. 1*C* for metachrony workflow example). ROI was analyzed for ciliary motion in phase. Each video was assessed for the presence of cilia in motion. Areas of ciliary activity were determined from µOCT images by identifying regions of the image with periodic oscillations in intensity that exceed random variations due to noise, defined for this purpose as four times the median standard deviation of each pixel in the image. Cilium tips are detected as high-intensity aggregated point scatters via A-lines from vertical scanning. The location of the tip varies across the ciliary stroke cycle- i.e., when in effective stroke, the cilium is fully extended above the apical surface compared with a lower position in the recovery stroke. The Matlab script assigns the cilia across the epithelium into binary green and red based on their relative positions in the power stroke or recovery/rest phase based on a 5 µm cutoff.

**Figure 2.**
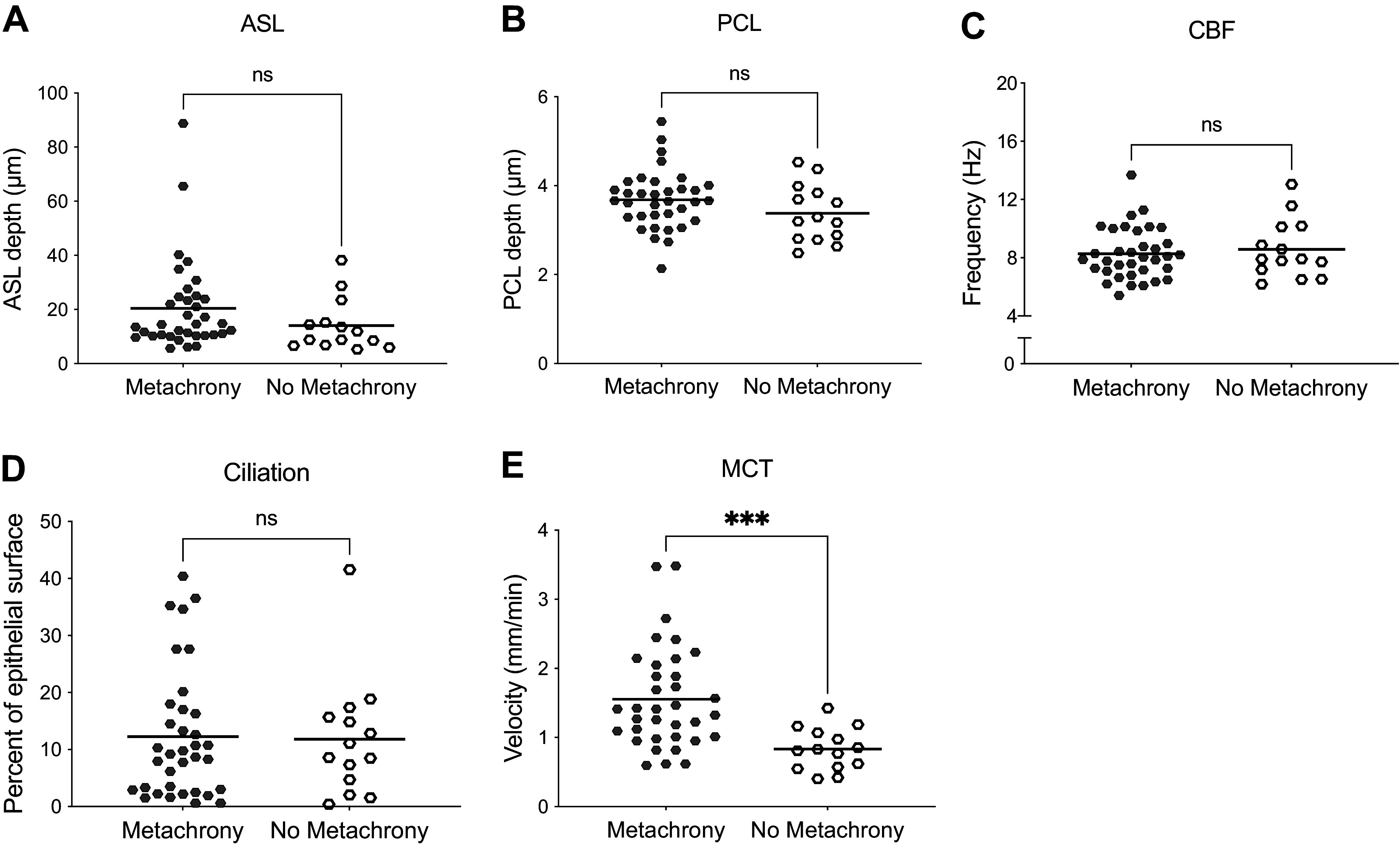
Mucociliary transport rate is bolstered when metachrony is present in human bronchi. Airway surface liquid depth (ASL; *A*), periciliary liquid depth (PCL; *B*), ciliary beat frequency (CBF; *C*), percentage of epithelial surface with detectable motile cilia (ciliation; *D*), and mucociliary transport rate (MCT; *E*) were quantified for each region of interest (ROIs). *n* = 35 metachrony ROIs and 14 no metachrony ROIs from eight distinct donors, ***P = 0.0009, as assessed by unpaired Mann–Whitney.

For metachrony characterization, a phase analysis of ciliary motion was measured with respect to the ciliary phase. Following the Fourier transformation of the µOCT signals, a MATLAB script encoded 0° and 180° phase shifts of ciliary stroke with respect to a reference cilium in green and red, respectively. The reference cilium was selected as the cilium with the sharpest peak from the CBF analysis at a single point in time. The color intensity of each pixel was scaled to the magnitude of the correlation between its complex vector and that of the reference cilium. The resultant image depicts the pattern of the ciliary phase of the epithelium. Red and green bands along the *X*-*Y* plane indicated areas of synchronous ciliary motion. Pixels without discernable ciliary motion (i.e., weak intensity below a threshold) were not examined further. Metachrony was defined when two factors were present in the ROI: *1*) at least 25% of the epithelial surface demonstrated a line of synchronous beating as evidenced by linear monochromatic bands (Fig. 1*B*), and *2*) these linear bands alternated between phases when analyzed over time (see Fig. 1, *D* and *E*, and Supplemental Fig. S1; https://doi.org/10.5281/zenodo.11060571). In contrast, areas without synchrony (i.e., red and green speckling) or coordinated phase shifts over time were nonmetachronous (see Fig. 1*A*). To quantify the degree of metachrony, metachrony proportion was defined as the percentage of epithelial surface by length.

### Statistics

All measurements were analyzed by an investigator blinded to the treatment of the trachea. All data are presented as the mean [95% CI]. Each tracheal sample data point represents the means of 5–10 ROI from the sample. Multiple linear, backward, and stepwise regression was used to determine whether ASL, PCL, CBF, metachrony, ciliation, and metachrony proportion were predictive of MCT rate; CBF*Metachrony was included as an interaction term based on biological plausibility. Comparisons of CBF and MCT in the presence or absence of metachrony were compared using unpaired *t* tests. Analysis of variance (ANOVA) was used to compare CBF, MCT, and metachrony proportion between control, drug (BAPTA-AM, LaCl_3_, or repaglinide) using variates, which were significantly predictive of the dependent variable based on multiple regression analysis. Post hoc comparisons were using the Brown-Forsythe test (due to unequal standard deviations across groups) in cases of significant omnibus Welsh ANOVA findings. Statistical analyses were conducted using GraphPad Prism 9.1.1 (GraphPad Software, Inc., La Jolla, CA) and IBM SPSS Statistics version 25 (IBM Corporation, Chicago, IL). Statistical significance for inferential comparisons was defined a priori as *P* < 0.05.

## RESULTS

The motion of motile cilium on a group of multiciliated cells can be characterized by an effective power stroke (green) and a recovery stroke (red) (Fig. 1, *inset*). There are three possibilities for ciliary intercoordination of these groups of motile cilia: *1*) groups of cilia beat independently, in a random, chaotic matter in which the stroke phase of one cilium does not influence the phase of its neighbors; *2*) cilia beat completely synchronously across a sizeable spatial dimension; or *3*) cilia beat metachronously in which the spatial-temporal characteristics of the ciliary cycle are coordinated slightly out of phase, generating an additional superimposed waveform. Figure 1 is a schematic diagram showing chaotic beating versus metachrony. A metachronal wave is defined in two dimensions by a line of synchronization and a perpendicular line of metachrony over the ciliary plane (16, 34, 35); the grid demonstrates this concept in Fig. 1*B* in which the numerical sequences correspond to the phase of the ciliary stroke. Figure 1*A* and Supplemental Fig. S1 show examples of chaotic motion, where several CBFs are represented in a small surface area (represented by color encoding CBF), compared with a region in metachrony with uniform CBF (Fig. 1*B*) and lines of synchrony that shift in space (Fig. 1*D*) and time (Fig. 1*E* and Supplemental Fig. S1). Figure 1*C* demonstrates the metachrony workflow for these analyses.

To evaluate the impact of metachrony, we used μOCT to visualize the functional microanatomy including the presence and proportion of epithelium in metachrony (defined dichotomously based on ciliary beat phase; see methods) of 17 airway segments (either main stem or B3) from eight distinct failed human donors, yielding 49 regions of interest (ROIs). Analysis was conducted on the pooled dataset. Metachrony was present in 47% of the ROIs, whereas others had chaotic motion. We compared ASL, PCL, CBF, proportion of the epithelium that had detectable ciliary motion, and MCT in areas with and without metachrony present. ASL depth was not significantly changed when metachrony was present compared with regions without metachrony (mean [95% CI] - 17.0 µm [13.7, 20.3] metachrony vs. 12.2 µm [7.8, 16.4] no metachrony, *P* = 0.0991; Fig. 2*A*). Similarly, PCL depth was not different in areas with metachrony compared with areas without metachrony (3.7 µm [3.5, 3.9] metachrony vs. 3.4 [3.0, 3.8] no metachrony, *P* = 0.1446; Fig. 2*B*). Mean CBF was no different in areas with or without metachrony (8.3 Hz [7.7, 8.9] metachrony vs. 8.6 Hz [7.4, 9.7] no metachrony, 0.5930; Fig. 2*C*). The ciliation (degree of detectable motile cilia per given area) was no different in areas with or without metachrony (12.3% of epithelial surface [8.4, 16.2] metachrony vs. 11.8% [5.8, 17.8] no metachrony, *P* = 0.9178; Fig. 2*C*). However, the impact on MCT was substantial, as MCT was 48.1% faster when metachrony was present compared with regions without metachrony (1.6 mm/min [1.3, 1.8] metachrony vs. 0.83 mm/min [0.7, 1.0] no metachrony; *P* = 0.0009; Fig. 2*E*).

To further discern the effects of metachrony, we developed a statistical model that included the covariates of MCT measured simultaneously and in a colocalized fashion by µOCT to control for these interrelated components of the mucociliary transport apparatus. Univariate analysis of each variable considered is shown in Table 1. In human bronchial explants, ASL, PCL, Metachrony, and Metachrony*CBF interaction were significantly associated with MCT. Next, to assess whether metachrony was independently important to augmenting MCT while controlling for other factors, which were also significantly predictive of MCT, we performed multiple linear, backward stepwise regression analysis. The final model included Metachrony and PCL (*R*^2^ = 0.189, *P* < 0.001) (Table 1). In human bronchial explants, the effect of metachrony on MCT remained significant (B = 0.471; *P* = 0.018) even when controlled for airway hydration by PCL depth (*P* < 0.009).

**Table 1.** Human univariate and multivariate analyses

Human Univariate Analysis				
	β Unstandardized	Coefficient of Error	β Standardized	*P* Value
ASL	**0.019**	**0.007**		**0.007**
CBF	0.080	0.053		0.136
Metachrony	**0.592**	**0.192**		**0.003**
PCL	**0.483**	**0.140**		**0.001**
Metachrony Length Ratio	0.253	0.444		0.571
Metachrony*CBF	**0.083**	**0.022**		**<0.001**

Human Multivariate Analysis				
	β Unstandardized	Coefficient of Error	β Standardized	*P* Value
Metachrony	0.471	0.195	0.257	0.018
PCL	0.388	0.144	0.285	0.009

Univariate analysis demonstrates contributors to mucociliary clearance rates in human bronchial explants. ASL depth, PCL depth, and Metachrony independently contribute to faster MCT. In multivariate analysis, multiple linear backward stepwise regression analysis identified independent contributors to mucociliary transport rates in human bronchial explants. The final model included Metachrony and PCL as predictors which contribute to MCT (*R^2^* = 0.189, *P* < 0.0001). ASL, airway surface liquid; CBF, cilia beat frequency; PCL, periciliary liquid. Bold values are statistically significant.

**Figure 3.**
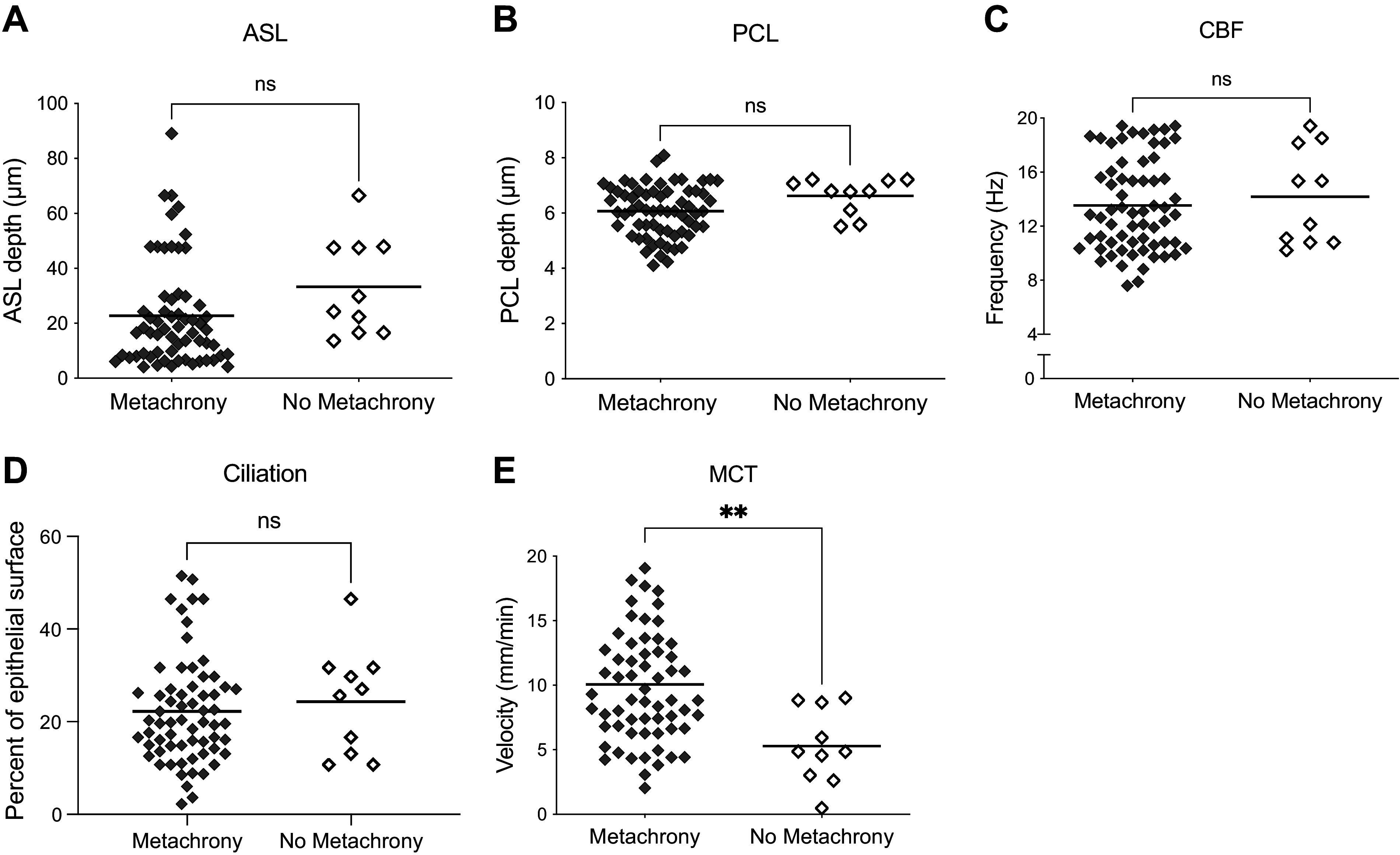
Ferret tracheae demonstrate faster mucociliary transport rate areas with metachrony. Airway surface liquid depth (ASL; *A*), periciliary liquid depth (PCL; *B*), ciliary beat frequency (CBF; *C*), percentage of epithelial surface with detectable motile cilia (ciliation; *D*), and mucociliary transport rate (MCT; *E*) were quantified for each region of interest (ROI). *n* = 62 metachrony ROIs and 10 no metachrony ROIs from 14 distinct ferrets, **P = 0.0020, as assessed by unpaired Mann–Whitney.

Given the limited availability of failed human bronchial explants, we wanted to confirm findings in a controlled animal model. We examined 74 ROIs from untreated trachea segments derived from 14 distinct ferret tracheae. Metachrony was present in 82% of the untreated ferret ROIs. We compared ASL, PCL, CBF, and MCT in areas with and without metachrony present and reported individual ROIs in Fig. 3. ASL depth was not significantly different when metachrony was present compared with regions without metachrony (22.8 µm [17.9, 27.6] metachrony vs. 33.2 µm [20.4, 46.0] no metachrony, *P* = 0.1085; Fig. 3*A*). PCL depth was not changed significantly deeper in areas with metachrony compared with areas without metachrony (6.4 µm [5.8, 6.3] metachrony vs. 6.6 [6.2, 7.1] no metachrony, *P* = 0.0723; Fig. 3*B*). CBF was not significantly faster in areas with metachrony compared with areas without metachrony (13.5 Hz [12.7, 14.4] metachrony vs. 14.18 Hz [11.6, 16.7] no metachrony, *P* = 0.5867; Fig. 3*C*). The ciliation was no different in areas with or without metachrony (22.3% of epithelial surface [19.4, 25.1] metachrony vs. 24.3% [16.1, 32.6] no metachrony, *P* = 0.54; Fig. 3*D*). MCT was 47.5% faster when metachrony was present compared with regions without metachrony in ferret tracheae (10.1 mm/min [8.9, 11.2] metachrony vs. 5.3 mm/min [3.2, 7.3] no metachrony; *P* < 0.0020; Fig. 3*E*).

As in human trachea, we performed a regression analysis to establish the independent contribution of metachrony to MCT. First, univariate analysis with MCT as the dependent variable was conducted with input variables ASL, CBF, Metachrony, PCL, Metachrony Length Ratio, ciliation, and the Metachrony*CBF interaction. In ex vivo ferret tracheae (all *P* < 0.05; Table 2), ASL, CBF, Metachrony, and the Metachrony*CBF interaction were independently identified as significantly predictive of MCT. Thus, we conclude that in both human and ferret airways, ASL and Metachrony are key independent contributors to the MCT rate, while PCL and CBF appear to influence human and ferret MCT rates, respectively.

**Table 2.** Ferret univariate and multivariate analyses

Ferret Univariate Analysis				
	β Unstandardized	Coefficient of Error	β Standardized	*P* Value
ASL	**0.056**	**0.027**		**0.038**
CBF	**0.558**	**0.174**		**0.002**
Metachrony	**5.153**	**1.754**		**0.004**
PCL	1.042	0.696		0.139
Metachrony Length Ratio	2.606	3.568		0.468
Ciliation	0.038	0.056		0.494
Metachrony*CBF	**0.468**	**0.099**		**<0.001**

Ferret Multivariate Analysis				
	β Unstandardized	Coefficient of Error	β Standardized	*P* Value
ASL	0.048	0.024	0.205	0.055
CBF	0.516	0.164	0.327	0.002
Metachrony	5.859	1.602	0.372	<0.001

Univariate analysis demonstrates contributors to mucociliary clearance rates in ferret tracheae. ASL depth, CBF, and Metachrony independently contribute to faster MCT. In multivariate analysis, multiple linear backward stepwise regression analysis identified independent contributors to mucociliary transport rates in ferret tracheae. The final model included ASL depth, metachrony, and CBF as predictors that contribute to MCT (*R^2^* = 0.287, *P* < 0.001). ASL, airway surface liquid; CBF, cilia beat frequency; PCL, periciliary liquid. Bold values are statistically significant.

In ferret tracheae, we found the effect of metachrony on MCT to be highly significant even when controlled for CBF and ASL (*P* < 0.001, Table 2). The final model included ASL, CBF, and Metachrony (*R*^2^ = 0.287, *P* < 0.001). MCT rate was positively correlated with ASL depth (Pearson’s correlation *r* = 0.242, *P* = 0.019), CBF (*r* = 0.354, *P* = 0.001), and Metachrony (*r =* 0.327, *P* = 0.002).

**Figure 4.**
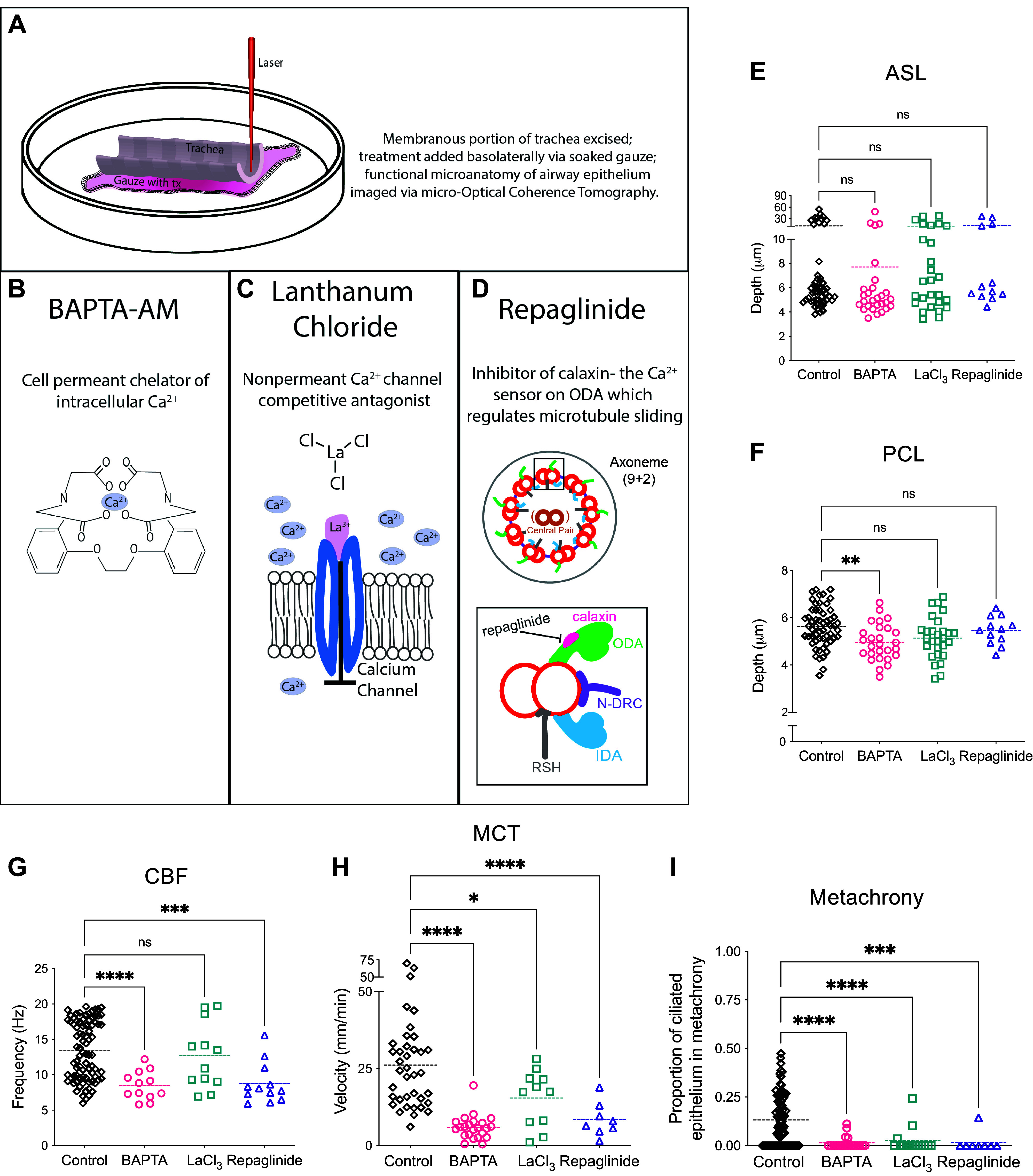
Pharmacological modulation of intracellular Ca^2+^, mechanosensitive calcium channels, and calaxin abolishes metachrony in ferret tracheae. *A*: schematic of how airway samples are cut longitudinally along the posterior trachealis muscle to expose the luminal airway epithelial surface to the µOCT laser. Treatments were added basolaterally via soaked gauze. *B*: BAPTA-AM is a cell-permeant chelator of intracellular calcium. *C*: lanthanum chloride works as a nonpermeant calcium channel competitive antagonist. *D*: repaglinide is an inhibitor of calaxin, the calcium sensor on the outer dynein arm (ODA). Airway surface liquid depth (ASL; *E*), periciliary liquid depth (PCL; *F*), ciliary beat frequency (CBF; *G*), mucociliary transport rate (MCT; *H*), and proportion of ciliated epithelium (*I*) in metachrony were quantified for each region of interest (ROIs). Not significant (ns) *P* > 0.05; *P = 0.0388; **P = 0.0024, ***P < 0.001, ****P < 0.0001, as assessed by Brown-Forsythe and Welch ANOVA tests. IDA, inner dynein arm; N-DRC, nexin-dynein regulatory complex; RSH, radial spoke head.

After establishing the role of metachrony in MCT rates, we further explored the mechanism by which metachrony is generated. We hypothesized that metachrony is calcium-dependent and thus tested the effect of BAPTA-AM (cell-permeant chelator of intracellular Ca^2+^), lanthanum chloride (LaCl_3_, a nonpermeant Ca^2 + ^channel competitive antagonist), and repaglinide (inhibitor of the Ca^2 + ^sensor, calaxin, which regulates microtubule sliding via the outer dynein arm) in ferret tracheae (Fig. 4). We examined 185 ROIs derived from tracheae segments originating from 14 distinct ferrets. ASL was not significantly different in control compared with any of the treatment group tracheae (10.96 µm [7.78, 14.13] control vs. 7.71 µm [4.32, 11.10] BAPTA-AM vs. 10.02 µm [6.38, 13.67] LaCl_3_ vs. 11.65 µm [4.53, 18.77] repaglinide) (Fig. 4*E*). Treatment with BAPTA-AM resulted in significantly diminished PCL compared with control, but neither LaCl_3_ nor repaglinide influenced the periciliary liquid depth (5.62 µm [5.39, 5.85] control vs. 4.96 µm [4.65, 5.26] BAPTA-AM, *P* = 0.0026 vs. 5.14 µm [4.80, 5.48] LaCl_3_, *P* = 0.0628 vs. 5.46 µm [5.08, 5.83] repaglinide, *P* = 0.8178) (Fig. 4*F*).

BAPTA-AM and repaglinide significantly decreased CBF compared with control, whereas LaCl_3_ did not alter CBF (13.47 Hz [12.57, 14.37] control vs. 8.74 Hz [7.19, 9.76] BAPTA-AM, *P* < 0.0001 vs. 12.68 Hz [9.73, 15.63] LaCl_3_, *P* = 0.923 vs. 8.75 Hz [6.91, 10.59] repaglinide, *P* = 0.0003) (Fig. 4*G*). Each treatment significantly decreased MCT compared with control (26.16 mm/min [21.36, 30.95] control vs. 5.94 mm/min [4.24, 7.65] BAPTA-AM, *P* < 0.0001 vs. 15.45 mm/min [9.32, 21.57] LaCl_3_, *P* = 0.0195 vs. 8.45 mm/min [3.91, 12.98] repaglinide, *P* < 0.0001) (Fig. 4*H*). BAPTA-AM, LaCl_3_, and repaglinide all significantly diminished the proportion of the epithelium beating in metachrony compared with control (mean, [95% CI], Brown-Forsythe post hoc *P* value relative to control: 13.3% [10.38, 16.12] control vs. 1.51% [–0.09, 3.11] BAPTA, *P* < 0.0001 vs. 2.5% [–1.12, 6.20] LaCl_3_, *P* < 0.0001 vs. 1.79% [–2.4, 6.00] repaglinide, *P* = 0.0003) (Fig. 4*I*).

## DISCUSSION

Effective mucociliary transport is essential to maintain adequate airway health. Motile cilia represent a critical aspect of the mucus clearance apparatus, and the integrated function requires precise motion and coordination to propel mucus. Previously, our group had identified that the application of a mucus load across the airway surface led to a downstream increase in mucociliary transport speeds (27). Here, we evaluate the function of metachrony within the intact MCT apparatus for the first time in two mammalian species. Metachrony is present in human and ferret airways and metachrony is a key contributor to effective MCT. Furthermore, metachrony appears to enhance transport rates above that predicted by airway hydration or ciliary beat frequency. Thus, metachrony is an independent contributor to mucociliary clearance, allowing mucus transport speeds to exceed the rate that would be conferred by individual ciliary motion. This effect is likely to be most significant in especially long tracheas where ciliary intercoordination has the greatest opportunity to interact and would be an advantageous adaptation to resolve airway obstruction by improving MCT.

Cilia have previously been described as an organelle under paracrine calcium control due to the importance of calcium for regulating ciliary beating; therefore, we hypothesized that the metachronal wave might be affected by altering intracellular calcium concentrations. To test this, we evaluated the effects of three compounds that alter Ca^2+^ availability to the functional cilia: BAPTA-AM, lanthanum chloride, and repaglinide. BAPTA-AM is a cell-permeant chelator, which is highly selective for calcium over magnesium. BAPTA-AM can be used to control the level of intracellular calcium, and its metal binding is less sensitive to pH compared with other chelators. To further explore the potential for the mechanosensitive propagation of metachrony, lanthanum chloride, a nonspecific, Ca^2 + ^channel competitive antagonist that is not cell permeable, was used to block mechanosensitive calcium channels along the airway surface in excised ferret tracheas. We hypothesized that the pharmacological blockade of mechanosensitive calcium channels with lanthanum chloride would be another mechanism by which metachrony could be completely or partially abolished, with a consequent decrease in MCT rate.

To complement this, we also tested whether calaxin signaling contributes to the development of metachrony. Previous studies have demonstrated that calaxin binds to dynein motor protein and may change the microtubular sliding in the calcium-dependent propagation of flagellar bending in sperm (31, 32, 36). Therefore, we hypothesized that repaglinide would function as a calaxin inhibitor and alter the dynein motor protein function of cilia on the airway surface, leading to an impaired wave of metachrony across the airway surface.

Metachrony was significantly reduced following treatment with BAPTA-AM, lanthanum chloride, or repaglinide, with a substantial decrease in MCT rate in all treatment groups (by 77.3%, 40.9%, and 67.7%, respectively) compared with vehicle control ferret tracheae. BAPTA-AM and repaglinide significantly diminished CBF by 35.1% and 35.0%, respectively. However, lanthanum chloride did not significantly influence CBF (5.9%, *P* = 0.923), suggesting that its effects on MCT were disproportionately large as compared with altered CBF. Evaluating other covariates, airway hydration as measured by ASL depth and PCL depth was not affected by treatment with the more cilia-specific calcium antagonists repaglinide or lanthanum chloride, although PCL depth but not ASL depth was diminished by BAPTA-AM treatment, likely through inhibition of Ca^2+^-dependent chloride secretion (37). Overall, this indicates that pharmacological inhibition of metachrony had a pronounced effect on MCT, even when CBF and airway hydration were preserved, supporting the regression analyses that also indicated an independent effect of metachrony on MCT. Furthermore, metachrony was dependent on intact intracellular Ca^2+^ through pathways that activate mechanosensitive receptors within cilia and function through calaxin signaling.

Previous studies by our group and others have shown that mucociliary transport is controlled by an autoregulatory mechanism that is sensitive to the degree of mucus load encountered by the epithelium (11, 33, 38, 39). The data presented here further add to the hypothesis that suggests induction of metachrony is another mechanism that the airways can preserve homeostasis when MCT is challenged through the activation of Ca^2+^ pathways (23, 33, 40). The effect of metachronal waves on the flow of mucociliary transport in vivo may be influenced by epithelial properties that affect the hydrodynamic coupling of neighboring cilia, such as cilia spacing and the density of multiciliated cells (8, 9, 41). This observation is supported by findings from studies involving artificial magnetic ciliary systems (42–45). Other aspects of mucus properties, including pH and viscosity could be investigated as stimulators of metachrony for future studies.

We showed that metachronal waves augment MCT rates, which is likely important when the airway is partially obstructed or mechanical events occur to stimulate MCT. We also found that calcium-dependent pathways of ciliary motion initiate and propagate metachrony. Furthermore, we demonstrated that metachrony can be disrupted by the chelation of intracellular calcium, competitive antagonism of mechanosensitive calcium channels, and inhibition of calaxin. In addition, the manipulation of calcium signaling exerts a deleterious effect on the mucociliary transport apparatus by altering the hydration of the periciliary liquid, CBF, and MCT rate. The current study demonstrates the nuanced calcium environment influencing ciliary motion, highlighting the role of intracellular Ca^2+^, mechanosensitive calcium channels, and calaxin in the coordination of cilia and the ultimate propagation of metachrony.

## DATA AVAILABILITY

Data will be made available upon reasonable request.

## SUPPLEMENTAL MATERIAL

10.5281/zenodo.11060571 Supplemental Fig. S1: https://doi.org/10.5281/zenodo.11060571.

## GRANTS

Funding was provided by NIH (1K08HL138153-01A1 and 2P30DK072482-12) and Cystic Fibrosis Foundation (CFF) (Solomon 20Y0) and by R35 HL135816-04S1 (to S. M. Rowe), P30DK072482-12 (to S. M. Rowe), 5F31HL146083-02 (to J. E. P. Lever), 3T32GM008361-30S1 (to J. E. P. Lever), and 2T32HL105346-11A1 (to J. E. P. Lever).

## DISCLOSURES

No conflicts of interest, financial or otherwise, are declared by the authors.

## AUTHOR CONTRIBUTIONS

J.E.P.L., S.M.R., and G.M.S. conceived and designed research; J.E.P.L., K.B.T., S.S.H., R.-J.S., V.Y.L., S.M.R., and G.M.S. performed experiments; J.E.P.L., K.B.T., C.M.F., H.M.L., S.S.H., R.-J.S., V.Y.L., S.E.B., K.K.C., G.J.T., S.M.R., and G.M.S. analyzed data; J.E.P.L., K.B.T., C.M.F., H.M.L., S.S.H., R.-J.S., S.E.B., K.K.C., G.J.T., S.M.R., and G.M.S. interpreted results of experiments; J.E.P.L., K.B.T., S.M.R., and G.M.S. prepared figures; J.E.P.L., K.B.T., S.M.R., and G.M.S. drafted manuscript; J.E.P.L., C.M.F., G.J.T., S.M.R., and G.M.S. edited and revised manuscript; J.E.P.L., K.B.T., C.M.F., R.-JS., V.Y.L., S.E.B., K.K.C., G.J.T., S.M.R., and G.M.S. approved final version of manuscript.
